# Proteomic and experimental analyses reveal molecular signatures of flexural atopic dermatitis in antecubital and popliteal fossae and the therapeutic effect of Aida lotion

**DOI:** 10.3389/fimmu.2026.1783354

**Published:** 2026-03-31

**Authors:** Fangrong Liu, Min Li, Jian Gong, Junhong Ye, Shanyu Qiu, Ke Xu, Qiao Liu, Weiwei Wu

**Affiliations:** 1 Clinical School of Medicine, Jiangxi University of Traditional Chinese Medicine, Nanchang, China; 2 Department of Dermatology, The Fifth People’s Hospital of Hainan Province, Haikou, China; 3 Department of Dermatology, Affiliated Dermatology Hospital of Hainan Medical University, Haikou, China; 4 Department of Integrated Traditional and Western Medicine of Dermatology, Dermatology Hospital of Jiangxi Province, Nanchang, China; 5 Department of Dermatology, The Second Affiliated Hospital of Jiangxi University of Traditional Chinese Medicine, Nanchang, China

**Keywords:** Aida lotion, antecubital fossae, atopic dermatitis, popliteal fossae, proteomics, site heterogeneity

## Abstract

**Introduction:**

The mechanisms underlying the predilection of atopic dermatitis for the antecubital and popliteal fossae remain unexplored. This preliminary exploratory study aimed to characterize the proteomic features of typical flexural atopic dermatitis lesions using proteomics integrated with animal model validation and to clarify the therapeutic mechanisms of Aida lotion.

**Methods:**

We recruited five patients with atopic dermatitis. Skin scale samples were collected from the flexural side of the antecubital and popliteal areas as the observation group, while samples from the corresponding extensor sides were used as the control group. Stratum corneum specimens were analyzed using ultra-sensitive proteomic techniques and bioinformatics to screen for differentially expressed proteins. Key candidate proteins were then validated in an atopic dermatitis mouse model to further explore the therapeutic mechanisms of Aida lotion.

**Results:**

A total of 712 differential proteins were identified. GO analysis indicated enrichment in pathways related to DNA replication, proteasome regulation, and myosin V binding. KEGG pathway analysis highlighted the importance of the PPAR and MAPK signaling pathways in the site-specific prevalence of atopic dermatitis. Key differentially expressed proteins including CYP27A1, CPT1A, FABP5 in the PPAR pathway and MAP2K3, MAP2K1, HRAS in the MAPK pathway, showed the highest fold changes. Animal experiments showed that atopic dermatitis induction increased SCORAD scores, edema, and inflammatory infiltration. Additionally, the expression levels of CPT1A, FABP5, MAP2K3, MAP2K1, and HRAS were upregulated, whereas the expression of CYP27A1 was downregulated, which was consistent with the proteomic findings, indicating that these targets have been validated as key proteins associated with the molecular pathology of flexural atopic dermatitis lesions in antecubital and popliteal fossa regions. Treatment with Aida lotion reversed these changes by downregulating CPT1A, FABP5, MAP2K3, MAP2K1, and HRAS, and upregulating CYP27A1, thereby alleviating dermatitis symptoms and reducing inflammatory and mast cell infiltration.

**Discussion:**

These findings identify CYP27A1, CPT1A, FABP5, MAP2K3, MAP2K1, and HRAS are prominent molecular signatures of atopic dermatitis in the antecubital and popliteal fossae. Aida lotion exerts therapeutic effects by modulating these key proteins and associated pathways.

## 1 Introduction

Atopic dermatitis is a chronic inflammatory skin disorder characterized by recurrent eczematous lesions, intense pruritus, and a personal or family history of atopic conditions (1). Atopic dermatitis often begins in infancy or early childhood and may persist into adulthood. As of 2021, there were approximately 126 million individuals worldwide living with atopic dermatitis, and the prevalence continues to rise, imposing a significant economic and psychological burden across all age groups (2). The pathogenesis of atopic dermatitis is influenced by a complex interplay of multiple factors (1), and remains incompletely understood. As a highly heterogeneous skin disease, atopic dermatitis most commonly involves the flexural areas, such as the antecubital and popliteal fossae, which is a hallmark clinical feature (3, 4). However, there is still a lack of systematic research on the intrinsic mechanisms underlying the predilection of atopic dermatitis for these flexural sites. Proteomics as a high throughput analytical technology, enables the identification of disease associated protein signatures by comparing protein expression profiles across different samples. When integrated with bioinformatics analysis, it becomes possible to explore the functional roles of differentially expressed proteins in the pathogenesis and progression of disease.

Traditional Chinese medicine (TCM) has attracted growing interest in the treatment of atopic dermatitis due to its notable clinical efficacy and its capacity to act on multiple molecular targets and biological pathways. Among various traditional therapies, topical herbal treatments have shown particular advantages, including high selectivity, strong site-specific effects, and excellent patient compliance, especially in younger populations (5). These features have led to their widespread application in the clinical management of atopic dermatitis. Aida lotion is a standardized herbal formula developed by Professor Liu Qiao, based on extensive clinical experience. It consists of eight medicinal herbs Artemisia argyi, Rheum officinale, Senecio scandens, Dictamnus dasycarpus, Chrysanthemum indicum, Sophora flavescens, Kochia scoparia, and Smilax glabra. Aida lotion has been extensively utilized in clinical settings for the management of atopic dermatitis, where it has shown significant efficacy in alleviating cutaneous lesions and relieving pruritus. Nevertheless, despite its long standing application in clinical practice, the underlying pharmacological mechanisms by which Aida lotion exerts its therapeutic effects remain largely unclear and merit further indepth investigation.

In this preliminary exploratory analysis, we integrated proteomics and animal experiments to investigate the underlying mechanisms contributing to the predilection of atopic dermatitis for antecubital and popliteal fossa and to elucidate the intervention pathways and therapeutic targets of Aida lotion. These findings provide new insights into the mechanisms underlying the anatomical heterogeneity of atopic dermatitis and offers novel perspectives for the treatment of atopic dermatitis using TCM.

## 2 Materials and methods

### 2.1 Proteomics clinical study

#### 2.1.1 Study participants

Participants were recruited from the outpatient dermatology clinic of the Affiliated Hospital of Jiangxi University of TCM between April 2024 and July 2024. Five patients with atopic dermatitis were enrolled in a self-controlled study design, in which the lesional flexural areas of the antecubital and popliteal fossae served as the observation group, while the corresponding extensor areas were used as the control group. This intraindividual self-controlled design was adopted to minimize genetic and environmental background noise, focusing on the most pronounced molecular differences between the clinically affected flexures and unaffected extensors. This study was approved by the Medical Ethics Committee of the Affiliated Hospital of Jiangxi University of TCM (Ethical Review Number: JZFYLL202402230028). Written informed consent was obtained from all participants prior to their inclusion in the study. Guardians of all children were informed and consented before collecting information and clinical samples.

#### 2.1.2 Diagnostic criteria

The diagnosis of atopic dermatitis was based on the Williams criteria (3) and the Zhang criteria developed in China (6).

The Williams criteria require the presence of the major criterion and at least three of the following minor criteria. Major criterion: pruritus. Minor criteria (1): History of involvement of the flexural surfaces, including the antecubital fossae, popliteal fossae, anterior ankles, and neck (in children under 10 years of age, involvement of the cheeks is also included); (2) Personal history of asthma or allergic rhinitis (or a first degree relative with atopic disease in children under 4 years of age); (3) History of generalized dry skin in the past year; (4) Visible flexural eczema (facial, forehead, or extensor limb eczema in children under 4 years of age); (5) Onset of symptoms before the age of 2 years (applicable to patients older than 4 years).

The Zhang criteria require the presence of item 1 and either item 2 or 3:(1) Symmetrical eczema lasting for more than six months;(2) Personal and/or family history of atopic diseases, including eczema, allergic rhinitis, asthma, or allergic conjunctivitis;(3) Elevated total serum IgE and/or peripheral blood eosinophilia and/or positive allergen specific IgE (defined as level 2 or higher in allergen specific IgE testing).

#### 2.1.3 Inclusion criteria

Participants were eligible for inclusion if they met the following conditions: (1) Aged under 60 years; (2) Diagnosed with atopic dermatitis according to both the Williams criteria and the Zhang criteria; (3) Presence of at least one visible lesion in the flexural areas of both the antecubital and popliteal fossae, with no lesions in the corresponding extensor regions; (4) Willingness to participate in the study and provision of written informed consent (for minors, consent was obtained from legal guardians).

#### 2.1.4 Exclusion criteria

Participants were excluded if any of the following conditions were present: (1) Presence of other concomitant dermatological diseases; (2) Presence of skin infections or infectious diseases such as syphilis or tuberculosis; (3) Impaired or dysfunctional hepatic, renal, or cardiac organ systems; (4) Coexisting cardiovascular, cerebrovascular, hepatic, renal, circulatory system diseases, malignancies, or metabolic disorders; (5) Receipt of any treatment within the past month that may interfere with study outcomes, including anti-infective, anti-inflammatory, anticancer therapies, or biologics targeted agents; (6) Presence of other uncontrolled, clinically significant medical conditions; (7) Pregnancy, lactation, or planning for pregnancy (8); Any other condition deemed inappropriate for inclusion by the investigators.

#### 2.1.5 Sample collection

We collected all samples in a temperature and humidity controlled laboratory to ensure environmental consistency, and required participants to refrain from bathing or applying any skincare products and topical therapeutic medications within eight hours prior to sampling in order to minimize external interference. For each participant, we selected lesional sites located in the antecubital and popliteal fossae, along with corresponding non lesional sites on the extensor surfaces, as designated sampling areas. Using sterile blunt surgical blades, we gently scraped skin scale samples from each site, transferred the collected samples into sterile cryogenic tubes and labeled them with the sampling time, anatomical location and participant identification number after which the samples were immediately stored at −80 °C until further analysis.

#### 2.1.6 Proteomics materials and reagents

The iRT (indexed Retention Time) standard peptides (Catalog No. 1816351) was obtained from Biognosys. Mass spectrometry-grade water (Catalog No. W6-4) and acetonitrile (Catalog No. A998-4) were acquired from Thermo Scientific (USA). Chromatography-grade methanol (Catalog No.67-56-1) and formic acid (Catalog No. FCMA117-50) were purchased from CNW (Germany). Dithiothreitol (Catalog No. 1064272) was obtained from Adamas-beta (Shanghai, China). Sera-Mag SpeedBead carboxylate-modified magnetic beads (Catalog No. 45152105050250) were acquired from Cytiva (Marlborough, MA, USA). Phenylmethanesulfonyl fluoride (PMSF; Catalog No. ST507) was obtained from Beyotime Biotechnology (Shanghai, China).2.1.7 Protein Extraction, LC-MS/MS, and Data Processing.

We first performed protein sample pretreatment by adding an appropriate amount of sample to SDS lysis buffer supplemented with phosphatase inhibitors and the protease inhibitor PMSF, followed by rapid centrifugation to ensure complete cell lysis and protein stabilization. The supernatant was then subjected to ultrasonic disruption, after which we added prepared SP3 magnetic beads and 100% acetonitrile, allowing the mixture to incubate at room temperature to facilitate protein binding. After successive steps involving supernatant removal, incubation, reduction with DTT and alkylation with IAA in the dark, and enzymatic digestion, the processed samples were prepared for subsequent mass spectrometry analysis. Data independent acquisition mass spectrometry was then performed, during which the IRT standard was mixed with the peptide samples at a volume ratio of 1:20 to serve as an internal retention time reference. Peptide separation was conducted prior to mass spectrometric analysis using a TimsTOF HT mass spectrometer operating in diaPASEF mode to analyze the atopic dermatitis samples.

All raw mass spectrometry data were integrated and processed using the PASER software platform. Database searching and protein-level DIA quantification were performed against the UniProt Homo sapiens database. To ensure high quality quantification across the self-controlled pairs, raw protein intensities were log2-transformed and subjected to Global Median Normalization within the PASER/bioinformatics pipeline to account for systematic injection variations. To maintain data integrity in this high-dimensional dataset, proteins with more than 30% missing values in any group were filtered out. Remaining missing values were addressed using k-nearest neighbors (KNN) imputation to prevent bias in subsequent differential expression analysis. For protein and peptide identification, the False Discovery Rate (FDR) was strictly controlled at < 0.01.

### 2.2 Animal experimentation

#### 2.2.1 Experimental animals

A total of forty SPF grade male Balb/c mice, aged 8 weeks with an average body weight of approximately 20 grams were obtained from corues biotechnology, SCXK (Jing) 2024-0001, Nanjing, China, and housed in the animal facility at the animal experimental center of Jiangxi University of TCM, SYXK (Gan) 2021-0004, Nanchang, China. Animals were randomly maintained under controlled environmental conditions with a room temperature ranging from 22 °C to 26 °C, relative humidity maintained between 40% and 60%, and a 12 hour light/dark cycle. All mice were subjected to a 7 day acclimatization period under standard feeding conditions before the start of the experiments. The study protocol was approved by the Animal Ethics Committee (Ethical Review Number: IACUC FJABR 2024101301).

#### 2.2.2 Drugs and reagents

Aida lotion (preparation code Z20210003000, batch number 20241010, specification 200 mL/bottle) was uniformly prepared and quality controlled by the Department of Pharmacy, The Fifth People’s Hospital of Hainan Province. The lotion is a standardized hospital preparation consisting of eight TCM (20 g each): *Artemisia argyi, Rheum palmatum, Senecio scandens, Dictamnus dasycarpus root bark, Chrysanthemum indicum, Sophora flavescens, Kochia scoparia fruit* and *Smilax glabra*. The lotion was prepared via standardized aqueous decoction and filtration, with the final product standardized to a crude drug content of 0.8 g/mL. Quality control was strictly maintained according to the Standardized Preparation Protocols for Hospital Medications, which include character identification and routine physicochemical inspections to ensure the stability and reproducibility of the clinical preparation. Eosin Y (Catalog No. D12621) was purchased from Xiya Reagent (Linyi, China). Hematoxylin (Catalog No. H9627-25G) was obtained from Sigma-Aldrich (USA). Toluidine blue (Catalog No. 71041284) was acquired from Wokai (Shanghai, China). The SDS-PAGE gel preparation kit (Catalog No. AS1012), RIPA total protein lysis buffer (Catalog No. AS1004), and BCA protein concentration assay kit (Catalog No. AS1086) were all purchased from ASPEN (Wuhan, China). TRIpure Total RNA Extraction Reagent (Catalog No. EP013) was obtained from ELK Biotechnology (Wuhan, China).

#### 2.2.3 Grouping, model induction, drug administration and tissue collection

Following a one week acclimatization period, forty SPF grade male Balb/c mice were randomly divided into four groups (n = 10 per group) using a random number table method: the normal group, the model group, the herbal medicine group and the positive group. The atopic dermatitis-like model was established based on a previously reported method (7). Specifically, 2,4-dinitrochlorobenzene (DNCB) was dissolved in a vehicle consisting of acetone and olive oil at a 4:1 (v/v) ratio to prepare 1.0% and 0.5% (w/v) working solutions. On day 0, a 2 cm × 3 cm area of dorsal hair was shaved to prepare a depilated region for induction. During the sensitization phase (days 1 to 3), 100 μL of 1% DNCB solution was topically applied and evenly distributed over the depilated skin area using the tip of a micropipette for all mice except those in the normal group. The application was conducted in a non-occlusive manner, allowing the solution to air-dry naturally to induce an atopic dermatitis-like inflammatory response. Beginning on day 8, the maintenance phase involved application of 100 μL of 0.5% DNCB solution every other day to sustain chronic skin inflammation. Acute phase (Day 1-7): Application of 1% DNCB to the dorsal skin induces significant inflammatory responses, including erythema, erosion, and exudation. Subacute and chronic phases (post day 7): Continuous intervention with 0.5% DNCB results in the development of dryness, thickening, and crusting, indicating successful model establishment.

**Figure 1:**
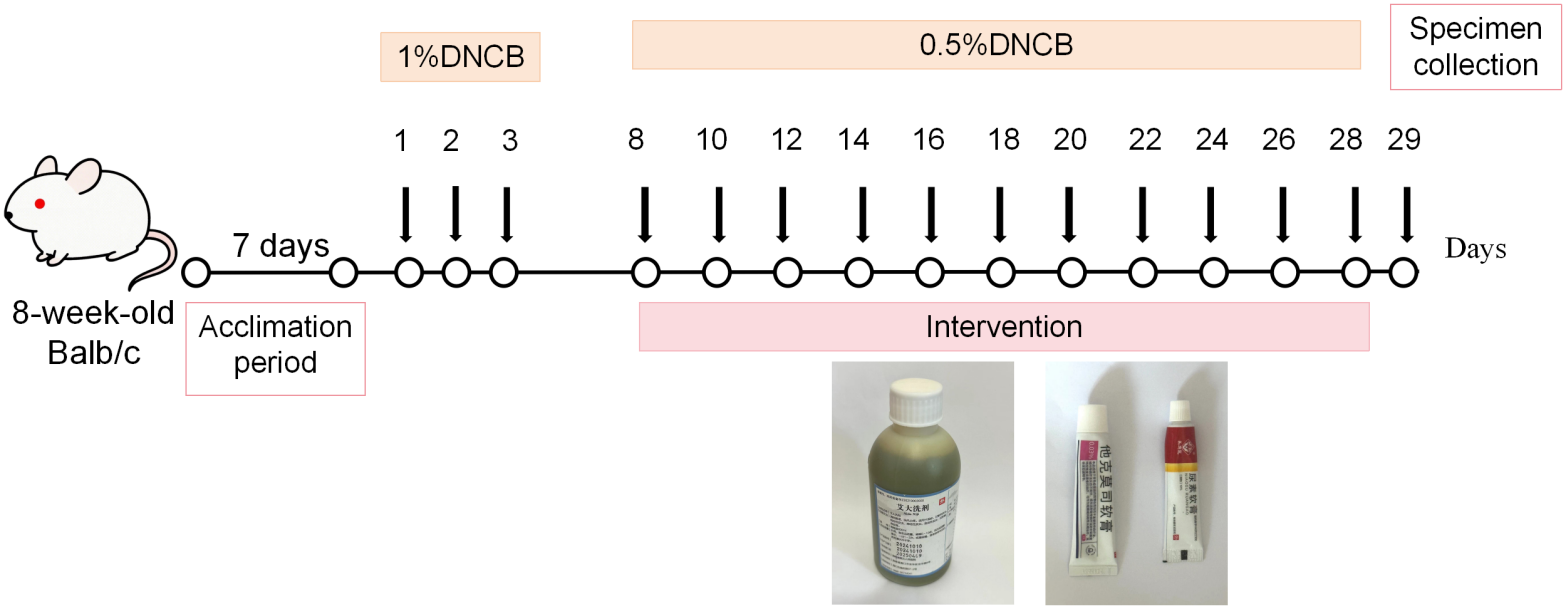
Schematic representation of the experimental design for the DNCB-induced atopic dermatitis mouse model.

From Day 8 to Day 28, the following interventions were applied: 1) Basic Treatment: All mice in the four groups received topical 10% urea cream twice daily as a standardized baseline regimen to simulate clinical skin barrier maintenance; 2) TCM Group: 30 minutes after urea application, the TCM group were treated with Aida lotion via wet compress, a characteristic external TCM therapy. The Aida lotion stock solution was freshly diluted 5-fold with sterile distilled water prior to each application. A sterile gauze (approximately 2 cm × 3 cm) soaked with the diluted Aida lotion was applied to the dorsal lesion, twice daily; 3) Positive group: After 30 minutes of urea ointment application, the mice were treated with tacrolimus ointment, twice daily. On day 29, all surviving mice were anesthetized with 1% sodium pentobarbital (50 mg/kg, i.p), and dorsal skin samples were surgically excised. Following tissue collection, the mice were euthanized by cervical dislocation, and death was confirmed by the cessation of heartbeat and respiratory arrest, consistent with ethical guidelines.A subset of three mice per group was randomly selected for downstream histological and molecular analyses to minimize experimental variance and adhere to the “Reduction” principle of the 3Rs in animal ethics. This selection was conducted using a random number table by a researcher blinded to the treatment groups (**Figure 1**).

#### 2.2.4 Observational indicators and methods

##### 2.2.4.1 Scoring of skin lesion severity

On days 0, 7, 14, 21, and 28 of the experiment, the severity of dorsal skin lesions in mice was evaluated using the Scoring Atopic Dermatitis (SCORAD) index. The total SCORAD score was calculated as the sum of the scores for erythema and hemorrhage, edema, excoriation and erosion, as well as dryness and scaling. Each parameter was assessed using a four point grading scale. For erythema and hemorrhage: 0 = none; 1 = mild erythema; 2 = pronounced erythema without hemorrhage; 3 = pronounced erythema with hemorrhage. For edema: 0 = none; 1 = mild; 2 = moderate; 3 = severe. For excoriation and erosion: 0 = none; 1 = mild excoriation; 2 = obvious excoriation without erosion; 3 = obvious excoriation with erosion. For dryness and scaling: 0 = none; 1 = mild dryness; 2 = obvious dryness without scaling; 3 = obvious dryness with scaling.

##### 2.2.4.2 Histological analysis

Fixed tissue specimens were subjected to a series of standard histological procedures, including embedding, sectioning, drying, dewaxing, and rehydration, to prepare paraffin embedded sections. These sections were subsequently stained with hematoxylin and eosin (HE) as well as toluidine blue.

To ensure objective quantification and reproducibility, three skin samples were randomly selected from each experimental group, and all evaluations were performed by two independent researchers blinded to the treatment groups. Specifically, the epidermal thickness was quantified by averaging measurements from three randomly selected, non-overlapping fields of view per section using digital image analysis software. Similarly, mast cell infiltration was quantified by counting toluidine blue-positive cells across three random high-power fields (HPF, 200× magnification) per slide. Data are expressed as mean ± standard error of the mean (SEM), and representative images were captured at 200× magnification.

##### 2.2.4.3 Quantitative PCR analysis of differential protein mRNA expression

Total RNA was extracted from skin tissue samples, and its concentration was quantified prior to reverse transcription into complementary DNA (cDNA) for subsequent amplification. Real-time PCR was performed under the following cycling conditions: initial denaturation at 95 °C for 30 seconds, followed by 40 cycles of denaturation at 95 °C for 10 seconds, annealing at 58 °C for 30 seconds, and extension at 72 °C for 30 seconds. Relative mRNA expression levels of target genes were calculated using the 2^-ΔΔCT^ method, with GAPDH serving as the internal control. To ensure data reliability, three animals per group were randomly selected (n=3, biological replicates), and each sample was analyzed in triplicate (technical replicates) to ensure measurement precision. The specific primer sequences, melting temperatures (Tm), and product lengths used in the reactions are summarized in (**Table 1**).

**Table 1:** Primer sequences and validation parameters for qPCR analysis.

Genes	Primer	Sequence (5′–3′)	Tm (°C)	GC%	Product length (bp)
GAPDH	sense	TGAAGGGTGGAGCCAAAAG	58.3	52.6	227
	antisense	AGTCTTCTGGGTGGCAGTGAT	58.4	52.4	
FABP5	sense	AGCTAGGAGTAGGACTGGCTCTTAG	59.7	52	134
	antisense	CAGGTTACAAGAGAACACAGTCGT	58.1	45.8	
MAP2K3	sense	CGGACCTTCATCACTATCGGA	59.9	52.4	253
	antisense	AGAGGGCACCATAGAAGGTGAC	59.8	54.5	
MAP2K1	sense	GCCTCTCAGCTCATATGGAATG	58.7	50	139
	antisense	GCATTTATTCACAAAATCCTGAAAC	56.2	32	
HRAS	sense	GACAGAATACAAGCTTGTGGTGG	59.1	47.8	155
	antisense	GTAGACATGTCTCCCCATCAATG	58.5	47.8	
CYP27A1	sense	CTGATAACCCTGGGATCCTACAT	59.1	47.8	128
	antisense	ATACTTCTGTACCAGCCTTGACAG	57.6	45.8	
CPT1A	sense	AGAGACTGTACGCTCCTGCACTA	58.8	52.2	107
	antisense	CACAAGCTATCTTGAACAGCTTG	57.5	43.5	

##### 2.2.4.4 Western blot analysis of differential protein expression

Total protein was extracted from skin tissue samples of each group, and protein concentrations were quantified using the bicinchoninic acid assay. Equal amounts of protein (10 microliters per lane) were subjected to SDS-PAGE, followed by electrotransfer onto PVDF membranes at a constant current of 300 mA for 90 min. The membranes were blocked with blocking buffer at room temperature for 1 hour. Subsequently, membranes were incubated with primary antibodies (detailed in **Table 2**) overnight at 4 degrees C. After washing, membranes were incubated with HRP-conjugated secondary antibodies (1:10,000, ASPEN) for 1 h at room temperature. The membranes were subsequently exposed in a darkroom with exposure conditions adjusted according to light intensity for development and fixation using an ECL kit (ASPEN). The films were scanned, and the optical density of target bands was analyzed using AlphaEaseFC software. Target protein levels were normalized to the loading control (GAPDH).

**Table 2:** Primary and secondary antibodies used for Western blot analysis.

Target protein	Host species	Vendor	Catalog No.	Dilution	Diluent
GAPDH (Control)	Rabbit	Abcam	ab181602	1:10,000	5% Non-fat milk
FABP5	Rabbit	Proteintech*	12348-1-AP	1:3,000	5% Non-fat milk
MAP2K3	Mouse	Proteintech*	68574-1-Ig	1:1,000	5% Non-fat milk
MAP2K1	Rabbit	CST	#12671	1:1,000	5% BSA
HRAS	Rabbit	Proteintech*	15531-1-AP	1:500	5% Non-fat milk
CYP27A1	Rabbit	Abcam	ab126785	1:2,000	5% Non-fat milk
CPT1A	Rabbit	Proteintech*	15184-1-AP	1:5,000	5% Non-fat milk
HRP-Goat anti Rabbit	Goat	ASPEN	AS1107	1:10,000	5% Non-fat milk
HRP-Goat anti Mouse	Goat	ASPEN	AS1106	1:10,000	5% Non-fat milk

"*" indicates the antibody manufacturer, where Proteintech refers to Proteintech Group, Inc. (Wuhan, China), and "#" denotes the catalog number of the antibody.

### 2.3 Statistical analysis

All statistical analyses were performed using GraphPad Prism 8.0 software and R (v4.2.1). Data were expressed as mean ± standard deviation (SD), and a P-value < 0.05 was considered statistically significant. For proteomics data, paired t-tests was used for comparisons meeting normality and homogeneity assumptions, while Wilcoxon signed-rank test was used when these assumptions were violated, with Benjamini-Hochberg FDR correction applied at q < 0.05. For the longitudinal lesion scores in animal experiments, two-way repeated measures ANOVA followed by Tukey’s *post-hoc* test was employed to compare differences across groups and time points. For multiple comparisons of other targets at single timepoints, one-way ANOVA followed by Tukey’s *post-hoc* test was utilized; in cases where data did not meet normality or homogeneity of variance assumptions, the Kruskal-Wallis test followed by Dunn’s *post-hoc* test was applied to adjust for multiple comparisons.

## 3 Results

### 3.1 Molecular signatures and protein dysregulation in flexural atopic dermatitis lesions

#### 3.1.1 Distinct proteomic landscapes between antecubital and popliteal lesions in atopic dermatitis

**Figure 2:**
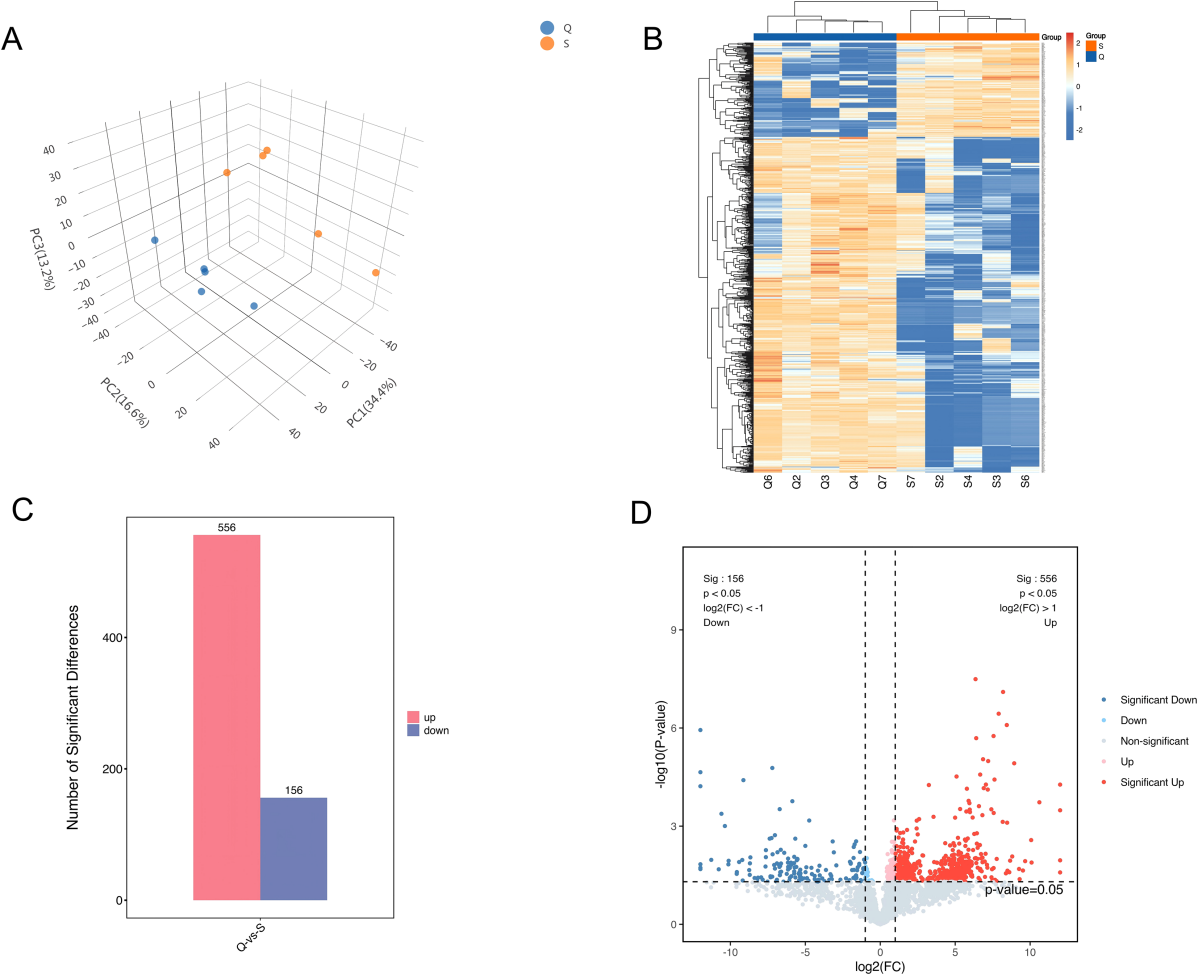
Representative proteomic analyses of differentially expressed proteins in the antecubital and popliteal fossa of atopic dermatitis. Q, observation group; S, control group. **(A)**. Principal component analysis; **(B)**. Heatmap of differentially expressed proteins; Red tiles indicate proteins with higher expression levels in the observation group (upregulated in Q relative to S), and blue tiles indicate lower expression levels (downregulated in Q relative to S), based on the calculation of log2(Q/S). **(C)**. Number of differentially expressed proteins; **(D)**. Volcano plot of differentially expressed proteins.

This study included a total of 5 patients with atopic dermatitis, consisting of 3 males and 2 females, with an average age of 8 ± 1.9 years (range: 6–11 years). To characterize the disease status at the time of sampling, clinical severity was evaluated by a dermatologist using the SCORAD index. The participants exhibited a mean SCORAD score of 34.6 ± 6.1 (range: 28.2 – 43.8), consistent with moderate atopic dermatitis. Although the initial inclusion criteria allowed for participants aged up to 60 years, all successfully recruited subjects with characteristic flexural lesions (antecubital and popliteal fossae) during the study period were within the pediatric age group. This distribution reflects the clinical reality that atopic dermatitis involvement in these specific flexural areas is most prevalent and characteristic in children. A total of 3,803 quantifiable proteins were identified through proteomic analysis. Principal component analysis (PCA) of protein quantification revealed clear separation between the two groups, indicating distinct differences in proteomic profiles; the greater the spatial distance between sample points, the more pronounced the intergroup differences (**Figure 2A**). Hierarchical clustering heatmap analysis further demonstrated significant differential protein expression between the observation group (Q) and the control group (S). In the heatmap, the x-axis denotes sample groupings, and the y-axis represents proteins with differential expression between the two groups. Each row corresponds to a specific protein, with red indicates upregulation in the observation group (calculated as log2(Q/S) > 0) and blue indicates downregulation (**Figure 2B**). Based on statistical criteria defined by a paired t-test with Benjamini-Hochberg correction (q < 0.05) and a fold change threshold of ≥2.0 or ≤0.5, a total of 712 differentially expressed proteins were identified, including 556 upregulated and 156 downregulated proteins in the observation group (Q) relative to the control group (S) (**Figure 2C, D**).

#### 3.1.2 Identification of PPAR and MAPK pathways as key drivers of flexural atopic dermatitis pathology

**Figure 3:**
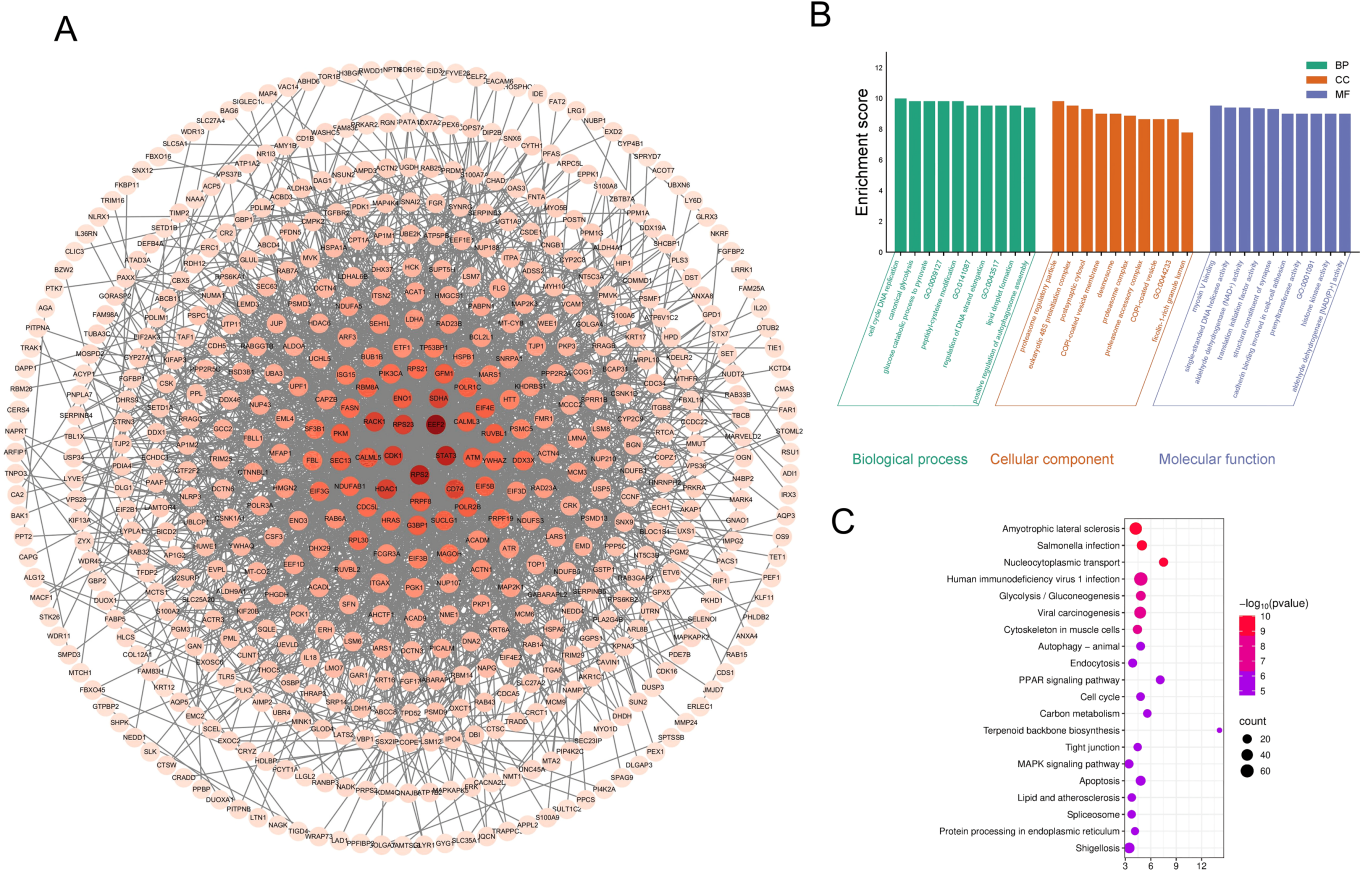
**(A)** PPI network of differentially expressed proteins; **(B)** GO analysis of differentially expressed proteins; **(C)** KEGG pathway enrichment analysis. GO: 0009127purine nucleoside monophosphate biosynthetic process; GO: 0009127purine nucleoside monophosphate biosynthetic process; GO: 0043517positive regulation of DNA damage response, signal transduction by p53 class mediator; GO:0044233mitochondria associated endoplasmic reticulum membrane contact site; GO: 0001091RNA polymerase II general transcription initiation factor binding.

Given the large number of differentially expressed proteins identified between the two groups, and in order to further elucidate the underlying mechanisms associated with the molecular characteristics of atopic dermatitis in the antecubital and popliteal fossae. The scope of analysis was narrowed to enable the precise identification of proteins associated with site-specific heterogeneity in atopic dermatitis. To this end, all differentially expressed proteins were imported into the STRING database. The interaction confidence score was set at 0.50 after testing different cutoffs. We found that higher thresholds (such as 0.70) caused the protein network to be too disconnected to show clear biological pathways, while 0.50 provided the best balance for identifying the key signaling clusters. This resulting in 560 protein–protein interaction data entries, which were subsequently visualized using Cytoscape version 3.9.1 (**Figure 3**). GO analysis revealed that in terms of biological processes (BP), the differentially expressed proteins were primarily involved in cell cycle DNA replication, canonical glycolysis, and glucose catabolic process to pyruvate. Regarding cellular components (CC), these proteins were predominantly localized to the proteasome regulatory particle, eukaryotic 48S preinitiation complex, and postsynaptic cytosol. For molecular functions (MF), the differentially expressed proteins were mainly associated with myosin V binding, single stranded DNA helicase activity, and aldehyde dehydrogenase (NAD+) activity (**Figure 3**). KEGG pathway analysis identified a total of 104 signaling pathways with statistical significance (*P* < 0.05), among which the top 20 were prioritized for presentation (**Figure 3**). The PPAR and MAPK signaling pathways ranked among the most enriched and were selected for further investigation. From each pathway, three highly upregulated differentially expressed proteins were chosen for validation: CYP27A1, CPT1A, and FABP5 from the PPAR pathway; MAP2K3, MAP2K1, and HRAS from the MAPK pathway.

### 3.2 Therapeutic efficacy of Aida lotion in an atopic dermatitis mouse model

#### 3.2.1 Aida lotion alleviates severity and skin lesion scores in atopic dermatitis mice

**Figure 4:**
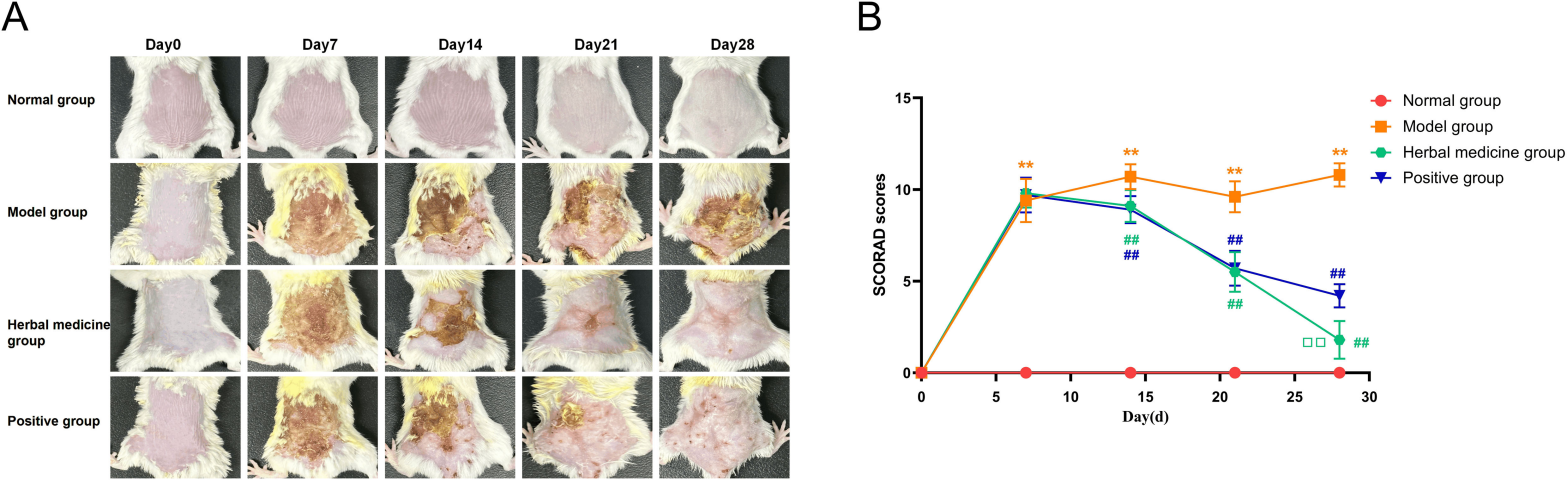
Aida lotion alleviate skin lesions induced by DNCB in atopic dermatitis mice. **(A)** Skin condition of mice in each group at different time points (n=10); **(B)** SCORAD scores of mice in different groups at various time points (n=10). Statistical significance was determined using two-way repeated measures ANOVA followed by Tukey’s multiple comparisons test for longitudinal SCORAD scores and skin thickness. Compared with the normal group, **P < 0.01; compared with the model group, ^##^*P* < 0.01;compared with the positive group, ^□□^*P* < 0.01.

Compared to the normal group, mice in the model group exhibited a significant increase in skin lesion scores (*P* < 0.01). The normal group showed smooth and even dorsal skin, while the model group developed distinct erythema with poorly defined borders and edema after three skin stimulations. By day 7, partial scab shedding exposed bright red erosive areas with exudation, and by day 14, dried exudate formed yellow scabs. At day 28, the skin became dry, rough, thickened, and covered with thick scabs, reflecting the clinical manifestations of atopic dermatitis. Compared to the model group, treatment groups demonstrated a trend of improvement in skin lesions, with significantly lower lesion scores on days 21 and 28 (*P* < 0.01). In both the herbal medicine group and positive groups, the dorsal skin lesions gradually resolved, with noticeable improvements in erythema, hemorrhage, and reduced edema. The original tearing and erosion improved, and dryness decreased. By day 28, the herbal medicine group showed more significant improvement compared to the positive group, with only mild dryness and desquamation remaining (*P* < 0.01). (**Figure 4**).

#### 3.2.2 Aida lotion suppresses inflammatory cell infiltration and mast cell recruitment in atopic dermatitis skin

**Figure 5:**
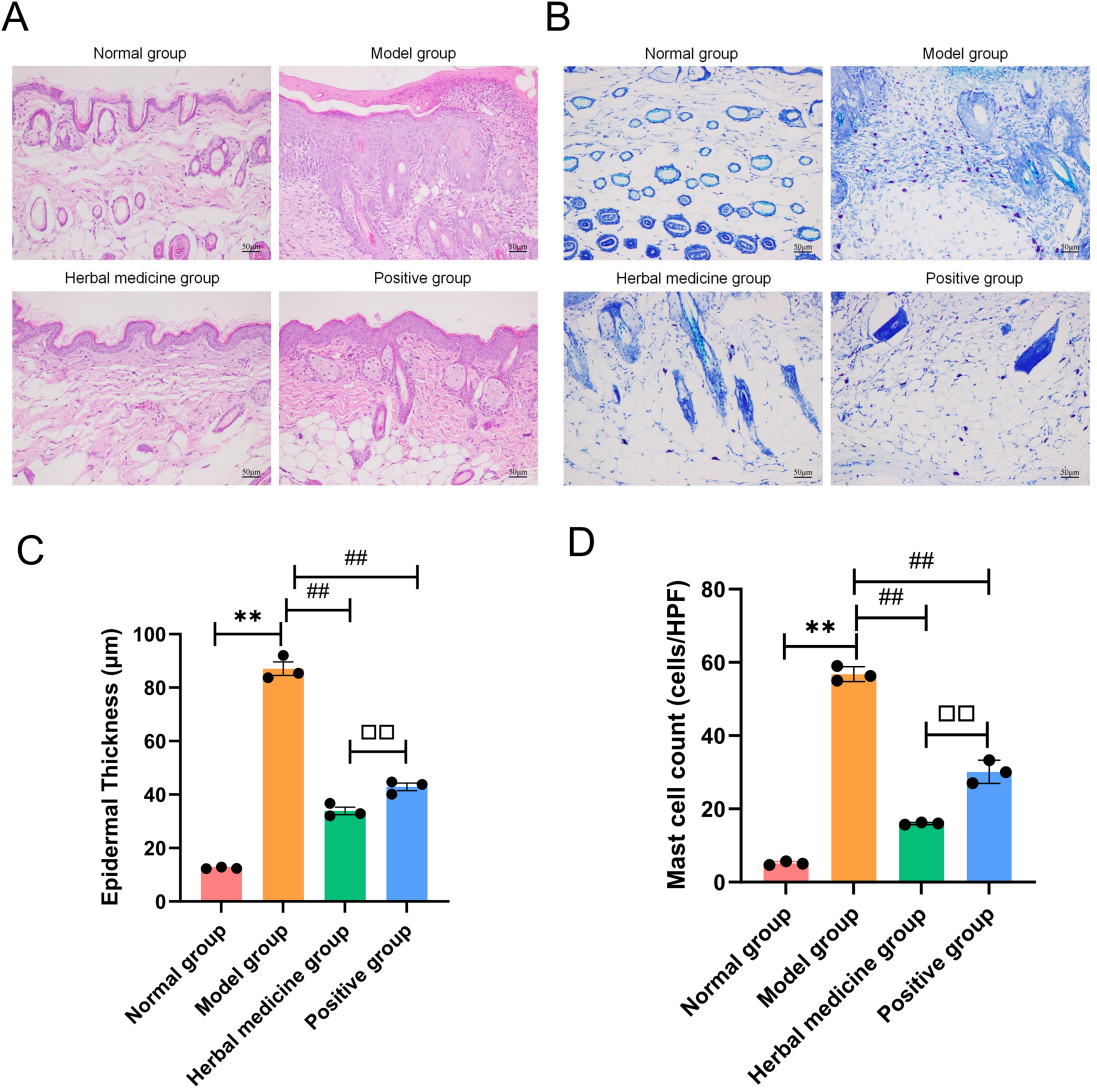
Histological staining of skin tissues from each group (HE, TB, ×200). **(A)** HE staining of skin sections from each group (n=3); **(B)** Toluidine blue staining of skin sections from each group (n=3); **(C)** Quantitative analysis of epidermal thickness (μm); **(D)** Quantitative analysis of mast cell counts per high-power field (HPF). Compared with the normal group, **P<0.01; compared with the model group, ^##^*P*<0.01; compared with the positive group, ^□□^*P*<0.01.

HE staining revealed that the dorsal skin of mice in the normal group exhibited normal structural integrity and cellular morphology, with a well-defined boundary between the epidermis and dermis, and no evidence of edema or inflammatory infiltration. In the model group, marked thickening of the stratum spinosum was observed, accompanied by both hyperkeratosis and parakeratosis. Intercellular edema was apparent within the epidermis, and substantial inflammatory cell infiltration was noted in the upper dermis. Compared with the model group, both the herbal medicine group and the positive group exhibited a reduction in inflammatory cell infiltration, including lymphocytes, along with alleviation of intracellular and intercellular edema; however, mild hyperkeratosis remained visible (*P* < 0.01). Notably, in comparison with the positive group, the herbal medicine group demonstrated more pronounced improvements, characterized by further reductions in both cellular edema and inflammatory infiltration (*P* < 0.01) (**Figure 5**). Toluidine blue staining revealed that a small number of mast cells were sparsely distributed in the skin of mice in the normal group. In contrast, the model group exhibited a dense infiltration of mast cells. Compared with the model group, the two intervention groups showed a marked reduction in mast cell infiltration, with a more pronounced decrease observed in the herbal medicine group (*P* < 0.01) (**Figure 5**). Quantitative analysis of epidermal thickness confirmed these morphological observations: the model group showed a significant increase in epidermal thickness compared to the normal group (*P* < 0.01), whereas Aida lotion treatment markedly attenuated this hyperplasia, resulting in a significantly lower epidermal thickness than that of the model group (*P* < 0.01; **Figure 5**). Similarly, quantitative counting of mast cells demonstrated that the dense infiltration in the model group was significantly reversed by Aida lotion. The reduction in mast cell density in the Aida lotion group was more pronounced than that in the positive control group (*P* < 0.01; **Figure 5**).

#### 3.2.3 Aida lotion modulates the transcription of key candidate targets within the PPAR and MAPK pathways

**Figure 6:**
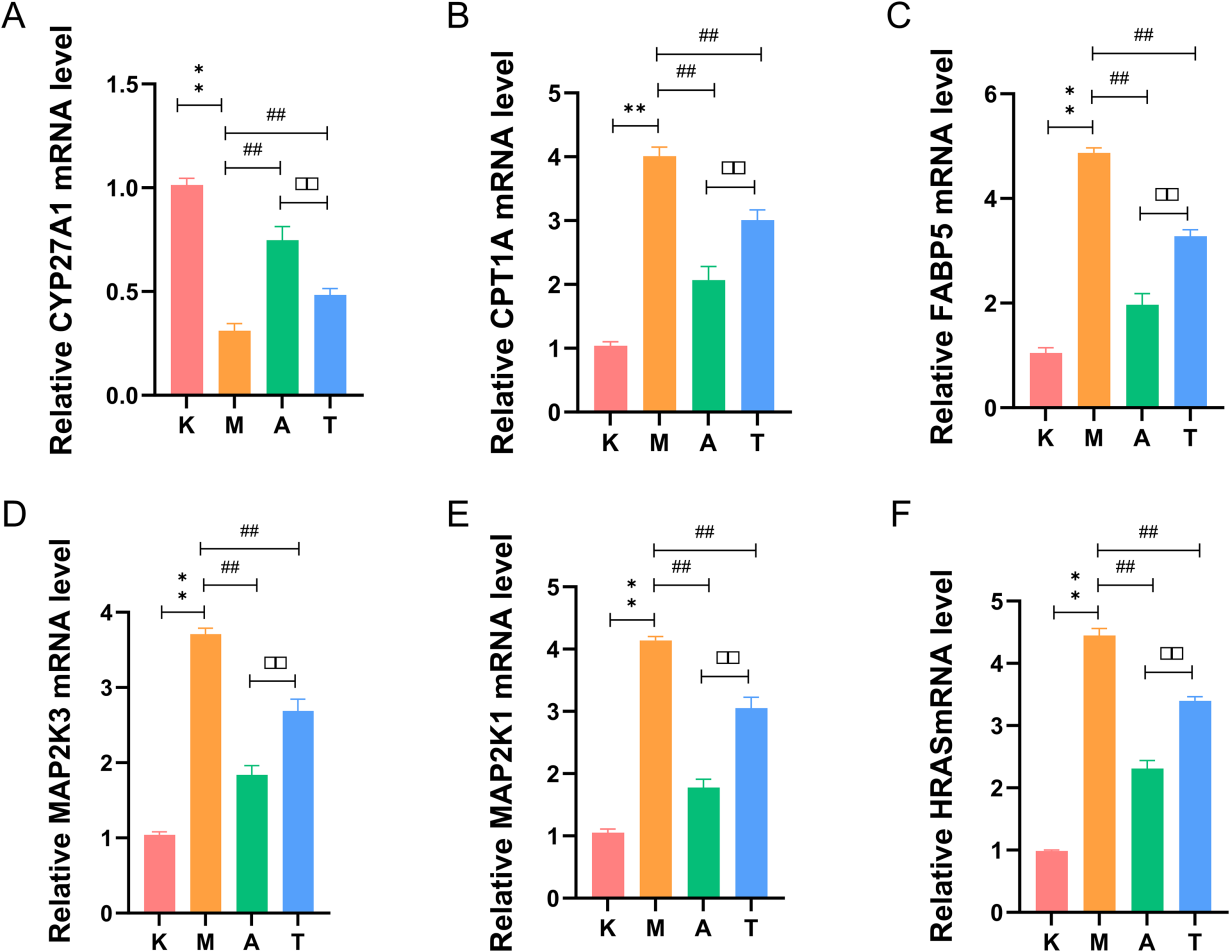
Relative mRNA expression levels of CYP27A1, CPT1A, FABP5, MAP2K3, MAP2K1, and HRAS (n = 3). **(A)** CYP27A1; **(B)** CPT1A; **(C)** FABP5; **(D)** MAP2K3; **(E)** MAP2K1; **(F)** HRAS. K: normal group; M: model group; A: herbal medicine group; T: positive control group. **P < 0.01 compared with the normal group; **P < 0.01 compared with the model group; ^##^*P* < 0.01 compared with the positive control group.

Based on the enrichment analysis of clinical proteomic data, we selected six representative differentially expressed proteins from the PPAR and MAPK pathways for validation in mouse skin tissues via RT-qPCR. Compared with the normal group, the model group showed significantly increased mRNA levels of CPT1A, FABP5, MAP2K3, MAP2K1, and HRAS in skin tissue (*P* < 0.01), while the level of CYP27A1 was significantly decreased (*P* < 0.01). In comparison with the model group, the herbal medicine group and positive group exhibited a significant upregulation of CYP27A1 mRNA and a significant downregulation of CPT1A, FABP5, MAP2K3, MAP2K1, and HRAS mRNA levels (*P* < 0.01), with the herbal medicine group showing a more pronounced reduction (*P* < 0.01) (**Figures 6A–F**). The mRNA expression trends of these targets were highly consistent with the initial proteomic findings, providing preliminary evidence of Aida lotion’s regulatory role at the transcriptional level.

#### 3.2.4 Protein level validation in AD mice confirms the regulatory impact of Aida lotion on targets identified via proteomics

**Figure 7:**
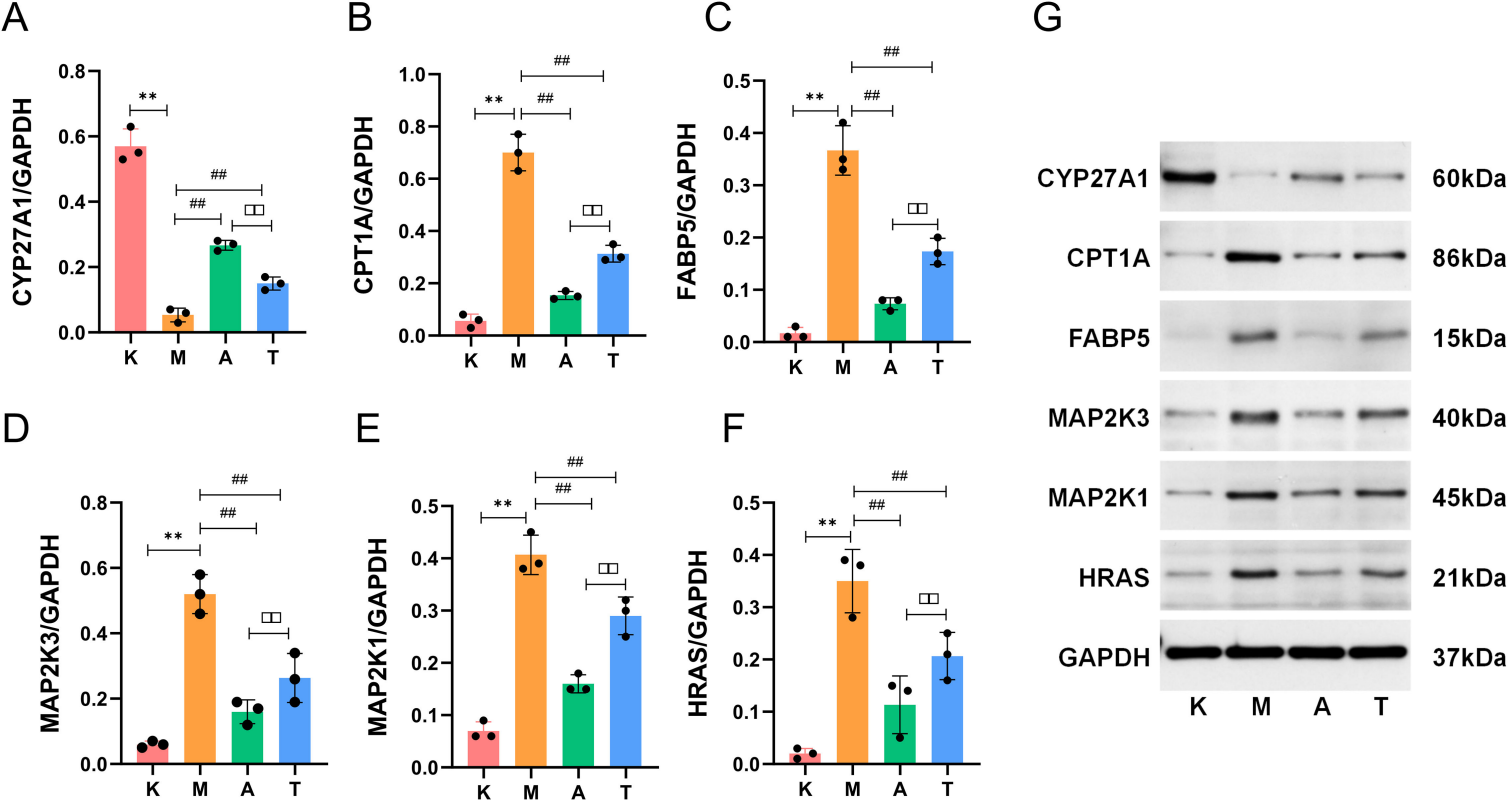
Protein expression levels of CYP27A1, CPT1A, FABP5, MAP2K3, MAP2K1, and HRAS. **(A)** CYP27A1/GAPDH; **(B)** CPT1A/GAPDH; **(C)** FABP5/GAPDH; **(D)** MAP2K3/GAPDH; **(E)** MAP2K1/GAPDH; **(F)** HRAS/GAPDH; **(G)** Representative Western blot images. Representative images were selected based on their proximity to the mean densitometric values of each group. All biological replicates (n = 3) were processed under identical experimental conditions, and the displayed blots reflect consistent trends across groups. K: normal group; M: model group; A: herbal medicine group; T: positive control group. **P < 0.01 compared with the normal group; **P < 0.01 compared with the model group; ^##^*P* < 0.01 compared with the positive control group; ^□□^*P* < 0.01.

To determine whether the protein expression patterns in the atopic dermatitis mouse model align with the molecular signatures identified in our clinical proteomic analysis, we evaluated the effects of Aida lotion on key targets using Western blot. Compared with the normal group, the model group exhibited significantly increased protein expression levels of CPT1A, FABP5, MAP2K3, MAP2K1, and HRAS (*P* < 0.01), while the protein expression level of CYP27A1 was significantly decreased (P<0.01). In comparison with the model group, both the herbal medicine group and the positive group showed a significant upregulation of CYP27A1 protein expression and a significant downregulation of CPT1A, FABP5, MAP2K3, MAP2K1, and HRAS protein levels (*P* < 0.01), with more pronounced changes observed in the herbal medicine group, which were statistically significant (*P* < 0.01) (**Figures 7A–G**). These results demonstrate that Aida lotion restores the expression of target proteins in a manner consistent with the proteomic profiles of AD lesions, confirming its efficacy in modulating key molecular drivers.

## 4 Discussion

Atopic dermatitis is characterized by pronounced heterogeneity across anatomical sites. Since the term was formally introduced in 1933 and defined as a condition associated with a family history of atopy, commonly preceded by infantile eczema, and exhibiting a characteristic pattern of lesion distribution (8), its predilection for specific anatomical locations has been a persistent focus of clinical interest. As widely accepted diagnostic cornerstones worldwide, the Hanifin and Rajka criteria (9) explicitly identify flexural involvement in adults and facial and extensor involvement in infants and young children as major diagnostic features, while the Williams criteria (3) incorporate a history of flexural involvement, including the antecubital fossae, popliteal fossae, and cervical region, into their core assessment framework. Subsequently, numerous authoritative guidelines, including the International Study of Asthma and Allergies in Childhood criteria (10), the Millennium criteria (11), the 2020 Japanese Dermatological Association guidelines (12), and the 2023 American Academy of Dermatology guidelines (4), have consistently emphasized involvement of flexural areas, such as the antecubital fossae, popliteal fossae, anterior knees, and neck, as a key clinical indicator for the diagnosis of atopic dermatitis. Nevertheless, existing studies on the anatomical distribution of atopic dermatitis have largely focused on epidemiological correlations involving age, sex, ethnicity, and geographic region.

Under the intertwined influence of genetic susceptibility and environmental exposures, epidermal barrier dysfunction and immune dysregulation are widely recognized as the principal mechanisms driving recurrent exacerbations of atopic dermatitis (4). As a highly heterogeneous dermatologic disorder, atopic dermatitis demonstrates a marked predilection in lesion distribution across anatomical sites, a phenomenon collectively shaped by epidermal barrier integrity, local microbial ecology, age at disease onset, and racial variation. Previous investigations have established that elevated transepidermal water loss represents a cardinal indicator of barrier impairment in atopic dermatitis. Choi et al. (13) reported that both intrinsic and extrinsic phenotypes exhibited significantly increased transepidermal water loss in the antecubital and popliteal fossae, whereas Agrawal et al. (14) demonstrated a substantial reduction in sebaceous lipid secretion compared with healthy controls. Furthermore, regional microenvironmental differences, including reduced microbial diversity and preferential colonization by Staphylococcus aureus within flexural areas (15), further amplify site specific susceptibility. The classical immunological framework posits that the stage dependent evolution of Th1 and Th2 immune responses constitutes the central axis of the atopic dermatitis disease course, with the acute phase characterized by Th2 predominant IgE mediated hypersensitivity and the chronic phase shifting toward Th1 driven cell mediated inflammation (16). This dynamic immunological transition closely parallels age dependent clinical transformation (17, 18), wherein lesions during childhood are predominantly confined to the face and flexural surfaces of the extremities, while during adolescence and adulthood the distribution progressively loses its flexural predominance and extensor surfaces become increasingly involved (19). In addition, geographic and ethnic variation warrants consideration, as Yew et al. (20) reported that flexural eruptions are particularly prevalent among East Asian and Southeast Asian populations. In the present study, proteomic profiling revealed that molecular divergence at predilection sites of atopic dermatitis is driven by intricate interactions among metabolic dysregulation, autophagy impairment, and inflammatory signaling. Collectively, the anatomical site preference of atopic dermatitis represents a multi-level, multi target regulated systemic process, in which the PPAR and MAPK signaling pathways are highly likely to function as central regulatory hubs that convert localized metabolic imbalance into clinically manifest inflammatory injury.

The DNCB-induced murine model was employed as a robust surrogate to establish a consistent inflammatory milieu characteristic of atopic dermatitis. While this model does not replicate the anatomical site-specificity of human flexural skin, it allows for the functional validation of candidate proteins and their response to therapeutic intervention within a generalized atopic dermatitis context. In animal experiments, Aida lotion demonstrated therapeutic effects, reducing SCORAD scores in mice while alleviating clinical manifestations including erythema, hemorrhage, edema, and crust formation. Histopathological evaluation further demonstrated that this formulation effectively mitigated epidermal hyperplasia and hyperkeratosis and attenuated inflammatory cell infiltration, with an efficacy comparable to that of the clinically established first line agent tacrolimus ointment. Notably, patients with atopic dermatitis frequently present with generalized xerosis in clinical settings, and emollients constitute a fundamental component of standard care that typically extends throughout the entire therapeutic course; accordingly, urea ointment was employed as a baseline moisturizer in the present study to recapitulate the clinically relevant combined treatment strategy of foundational hydration and active intervention. The therapeutic effects of Aida lotion were not confined to macroscopic symptom improvement but were further reflected in profound modulation of key molecular mechanisms, as evidenced by its capacity to restore imbalanced expression of proteins within the PPAR and MAPK signaling pathways identified through proteomic screening. In addition, toluidine blue staining revealed a pronounced reduction in mast cell infiltration, suggesting that Aida lotion may exert its distinctive anti allergic and anti-inflammatory effects through modulation of signaling cascades mediated by the high affinity IgE receptor (21). Given the high safety profile, favorable patient adherence, and low risk of adverse effects associated with topical traditional Chinese medicine formulations in infant and pediatric populations, this multi target and multi pathway regulatory paradigm provides robust scientific support for the long term management and precision intervention of anatomically susceptible sites in atopic dermatitis.

The proteomic samples in the present study were derived from epidermal scales collected from lesional skin at the antecubital and popliteal fossae of patients with atopic dermatitis; therefore, the identified differentially expressed proteins predominantly reflect pathophysiological alterations within the epidermal layer, particularly involving keratinocytes. Enrichment analyses indicated that dysregulation of the PPAR and MAPK signaling pathways may serve as key driving forces in the pathogenesis of atopic dermatitis at its characteristic predilection sites, namely the antecubital and popliteal fossae. Specifically, PPAR has been established as a critical positive regulator of keratinocyte differentiation. In particular, the widespread suppression of PPAR signaling undermines the synthesis of barrier-associated proteins such as filaggrin and impairs lipid metabolic integrity. This differentiation defect subsequently triggers compensatory keratinocyte hyperproliferation, which constitutes a pathological hallmark of atopic dermatitis and represents a key driver of the epidermal thickening observed in our clinical samples (22). Previous studies support this concept, demonstrating that epidermis specific deletion of PPARγ in mice results in spontaneous development of inflammatory skin lesions with features of atopic dermatitis, accompanied by pronounced upregulation of proliferation associated keratins Krt6, Krt16, and Krt17 (23), whereas topical administration of PPARα and PPARβ/δ agonists can effectively improve atopic dermatitis-like symptoms by promoting cornified envelope formation and improving skin barrier function (24, 25). In marked contrast, the MAPK/ERK pathway is highly activated in atopic dermatitis and is closely linked to aberrant keratinocyte proliferation. The ERK pathway is a major component of the MAPK cascade, which is highly activated in both mouse and human atopic dermatitis lesional skin, and this activation is closely linked to aberrant keratinocyte proliferation and epidermal hyperplasia (26). In atopic dermatitis lesions, keratinocyte proliferation markers such as Ki-67 are significantly upregulated compared to normal skin (27). Furthermore, a systematic review of keratinocyte activation mechanisms in inflammatory skin conditions has identified MAPK cascades as a recurring theme in atopic dermatitis pathogenesis, contributing to both epidermal hyperproliferation and barrier dysfunction (28). whereas pharmacological inhibition of this pathway has been shown to substantially ameliorate atopic dermatitis like pathology (29). Taken together, the pronounced dysregulation of the PPAR and MAPK signaling pathways identified in this study collectively reflects disruption of keratinocyte homeostasis within the epidermis of atopic dermatitis, which is tightly associated with the pathological features of epidermal hyperproliferation and defective differentiation.

PPARs comprise three subtypes: PPARα, PPARβ/δ, and PPARγ (30), Once activated by ligands, they form heterodimers with retinoid X receptors (RXRs) to initiate transcription, thereby exerting a variety of biological effects, including the regulation of immune responses to limit inflammation and the modulation of lipid metabolism (31). The expression of CYP27A1 is regulated by PPARγ and RXR, and this enzyme participates in the PPAR signaling pathway primarily by influencing cholesterol and lipid metabolism (32). Studies have shown that reduced levels of vitamin D increase the risk of atopic dermatitis in adults (33). The vitamin D–associated SNP rs4674343 in CYP27A1 has been identified as a protective factor against atopic eczema in the Chinese population (34). Suzuki et al. reported that rare variants of CYP27A1 significantly increase the risk of high IgE type atopic dermatitis in the Japanese population by disrupting the vitamin D metabolic pathway (35). Therefore, it is hypothesized that CYP27A1 may influence the predilection sites of atopic dermatitis lesions mainly through the vitamin D metabolic pathway. CPT1A is a downstream effector of the PPAR signaling pathway, which is a key enzyme involved in mitochondrial fatty acid oxidation (FAO). Although current research has primarily focused on its role in cancer, CPT1A has been shown to promote tumor progression by supporting energy production, resisting apoptosis, and facilitating epigenetic remodeling (36). In the context of immune and inflammatory diseases, elevated CPT1A expression can exacerbate conditions such as multiple sclerosis and experimental autoimmune encephalomyelitis by enhancing lipid metabolism, increasing oxidative stress, and promoting demyelination (37). However, a study by Wang et al. using a mouse model of acute lung injury revealed that CPT1A functions as a critical negative regulator of macrophage mediated inflammatory responses. It achieves this by suppressing glycolysis and promoting the polarization of macrophages toward the anti-inflammatory M2 phenotype, thereby exerting anti-inflammatory effects (38). Other studies have demonstrated that inhibition of FAO using CPT1A targeted inhibitors, in combination with the decoy effect of macrophage membranes and microneedle based delivery systems, can effectively provide synergistic therapeutic outcomes and prevent recurrence in psoriasis (39). Nevertheless, the role of CPT1A in the pathogenesis of atopic dermatitis remains unclear. Further investigation is required to elucidate the specific pathways through which CPT1A may contribute to the anatomical heterogeneity of atopic dermatitis lesions. FABPs located upstream of PPAR receptor activation, play essential roles in the regulation of lipid metabolism, gene expression, cell growth, and differentiation (40, 41). Among them, FABP5 is widely expressed in epidermal keratinocytes and immune cells such as T cells and macrophages, and is closely associated with the regulation of immune function and inflammatory responses (42). FABP5 regulates the proliferation and differentiation of keratinocytes in both healthy individuals and patients with psoriasis (43). In addition, it promotes NFκB signaling and neutrophil infiltration in psoriatic lesions (44). Elevated FABP5 expression in the skin of mice fed a high fat diet has been identified as a key factor contributing to diet induced skin inflammation (45). Furthermore, in a depilatory induced mouse model of dermatitis, FABP5 has been shown to promote cutaneous inflammation through lipid mediated mechanisms (46). Collectively, these findings suggest that FABP5 plays an important role in inflammatory pathways in the skin. However, the intrinsic link between FABP5 and atopic dermatitis remains unclear. It is speculated that FABP5 may influence the localization of atopic dermatitis lesions through metabolic and inflammatory pathways driven by excessive fatty acids.

The MAPK signaling pathway is an important mechanism involved in the pathogenesis of atopic dermatitis, participating in cellular pathological processes such as cell growth, differentiation, and inflammation. Its subfamilies p38, ERK, and JNK are all involved in the inflammatory process by regulating the expression of inflammatory cytokines and chemokines (47). MAP2K3 is located upstream in the MAPK signaling pathway and acts as a direct activator of p38MAPK. As a key epigenetic regulatory target of HDAC8 and HDAC9 in epidermal keratinocytes, MAP2K3 can amplify the inflammatory response by activating the p38 MAPK pathway. Its expression is elevated in inflammatory skin diseases such as atopic dermatitis, psoriasis, and acne (48, 49). In terms of treatment, MAP2K3 has been identified as a key target in peripheral blood mononuclear cells of patients with atopic dermatitis. Lower expression of MAP2K3 has been shown to better predict favorable therapeutic outcomes with dupilumab (49). MAP2K1 is an upstream kinase in the MAPK signaling pathway and primarily regulates cellular functions and inflammatory responses by activating the ERK pathway. Studies have confirmed that the ERK pathway can impair the skin barrier by suppressing proteins related to keratinocyte differentiation, while also promoting the recruitment of Th2 cells by inducing chemokines such as CCL17 and CCL22, thereby driving the progression of atopic dermatitis. Targeted inhibition of the ERK pathway has been shown to improve both skin barrier function and the inflammatory state in atopic dermatitis (26). However, current research directly investigating the relationship between MAP2K1 and skin diseases has mainly focused on melanoma (50), with few studies examining its role in atopic dermatitis. Further indepth exploration of the mechanisms involving MAP2K1 and the ERK pathway may provide a new direction for future studies on atopic dermatitis. HRAS is an upstream regulator in the MAPK signaling pathway and serves as a key modulator of the Ras-Raf-MEK-ERK cascade. It maintains skin homeostasis by dynamically balancing keratinocyte proliferation and senescence (51). Studies have shown that the G12S mutation in the HRAS gene leads to sustained activation of ERK signaling, which on one hand suppresses the expression of Claudin 1, a tight junction protein in the epidermis, and on the other hand induces excessive secretion of IL-33 by keratinocytes. This promotes the recruitment of type 2 innate lymphoid cells and drives Th2 type inflammatory responses, ultimately resulting in skin barrier dysfunction and atopic dermatitis-like lesions (52). Early reports indicated increased expression of HRAS in the skin of patients with psoriasis (53). Recent studies have found that HRAS G12S mosaic mutations in keratinocytes can continuously activate the MAPK and PI3K-Akt pathways, promoting abnormal keratinocyte proliferation and the release of inflammatory cytokines, thereby activating Th17 cells and leading to unilateral psoriatic lesions (54).Inhibiting the activity of the HRAS gene or its downstream signaling pathways may represent a potential therapeutic strategy for treating inflammatory skin diseases, including atopic dermatitis.

## 5 Conclusions

Through clinical proteomics and murine model validation, this preliminary exploratory analysis demonstrates that the PPAR and MAPK signaling pathways play pivotal roles in the molecular pathology of flexural atopic dermatitis lesions. Aida lotion exerts therapeutic effects by rebalancing the dysregulated expression of key targets within these pathways, including CYP27A1, CPT1A, FABP5, MAP2K3, MAP2K1, and HRAS. Although this study has provided preliminary insights, several limitations remain. First, The clinical comparison between lesional flexural and non-lesional extensor skin introduces a confounding factor between anatomical site effects and lesional inflammation; consequently, the identified differentially expressed proteins represent a combined molecular signature of both. Second, given the relatively small sample size, the potential risks of data overfitting and false-positive findings cannot be completely ruled out. Additionally, the exclusive use of a DNCB-induced murine model limit the generalizability of the findings. To address these issues, future validation in larger, multicenter cohorts across different age groups, alongside human ex vivo skin explants or 3D organoids, is required to disentangle site-specific predispositions from inflammatory responses, thereby enhancing the translational relevance of these findings for clinical application.

## Data Availability

The raw data supporting the conclusions of this article will be made available by the authors, without undue reservation.
